# The Effect of Mini Dental Implant Number on Mandibular Overdenture Retention and Attachment Wear

**DOI:** 10.1155/2023/7099761

**Published:** 2023-04-30

**Authors:** Rafif Alshenaiber, Craig Barclay, Nick Silikas

**Affiliations:** ^1^Prosthetic Dental Sciences Department at College of Dentistry, Prince Sattam Bin Abdulaziz University, Al-Kharj, Saudi Arabia; ^2^Oral Rehabilitation, University Dental Hospital of Manchester, Manchester, UK; ^3^Division of Dentistry, Faculty of Biology, Medicine and Health, University of Manchester, Coupland 3 Building, UK Manchester M13 9PL

## Abstract

**Purpose:**

Evaluate the effect of different mini-implant numbers on overdenture retention and evaluate attachment wear following one year of simulated placement/removal. *Material and Methods*. Nine models simulating atrophic mandibles held 27 mini dental implants in three groups of 2, 3, and 4 mini-implants. A total of 1080 simulated placement/removal cycles were carried out, and a digital force gauge was used to measure the overdenture dislodgment force. The means of the retention forces were analyzed using SPSS with one-way ANOVA and post hoc (*p* < 0.05). The inner diameter of attachment inserts was evaluated using a light microscope before and after testing. A paired *t*-test was used to compare the mean of inner ring diameters (*p* < 0.05).

**Results:**

The retention was significantly reduced regardless of the mini dental implant number, but the number affected overdenture retention. The placement of 4 mini dental implants provided higher retention and less reduction in retentiveness. However, no significant difference was found when 3 mini dental implants were compared to 2 mini dental implants (*p* = 0.21). Microscopic examination showed abrasion wear in all inserts following testing. However, the inserts of the 4 mini dental implants showed less wear than those used for 2 or 3 mini dental implants with *p* ≤ 0.001 and *p* ≤ 0.001, respectively.

**Conclusion:**

Mini dental implant overdenture retention force and attachment wear could improve by increasing the mini dental implants to 4. However, there was no difference in retention force or attachment wear when 2 or 3 mini dental implant overdentures were compared.

## 1. Introduction

There has been a consensus that implant overdentures should become the main treatment option to provide a retentive prosthesis for edentulous mandibles [1, 2]. Most studies measure patient satisfaction with the retentiveness of their overdentures [3, 4]. Mini dental implant (MDI) placement helps improve prosthesis retention for edentulous patients [5]. However, it is essential to address retention with MDI overdenture, especially in atrophic mandibles, where patients suffer the most from denture looseness [6–8].

Dental implants, including MDIs, can provide complete retention or partially share it with the residual ridge, depending on their number and prosthetic design [9]. Fixed prosthesis restoring edentulous mandible can be supported by 4 or 6 implants [10], while typically placing two implants in the mandible would help retain removable overdenture with mucosal support [1, 2]. With fewer implants needed for this treatment approach, it is easier to plan, cheaper to perform, and easier to maintain [11]. However, the diagnostic findings and patients' expectations determine the prosthesis type and implant number. Moreover, examination of the residual ridge is essential in determining the length and angulation of the implant [12].

Since their introduction as a prosthetic solution to retain overdentures, it has been recommended to use 4 MDIs in the mandible and 6 in the maxilla [13]. Therefore, some researchers followed the same recommendation with MDI numbers [14–18]. This arrangement was on the basis that the increased number would compensate for the reduced diameter and smaller implant/bone contact area. Therefore, one study evaluated the clinical survival rate of overdentures supported by different numbers of MDIs [19]. Moreover, increasing MDI number was associated with higher mechanical stability for overdenture [20].

On the other hand, others attempted to utilize the two-implant standard of care in the MDI treatment approach [5, 21, 22]. However, providing overdenture with the minimum number of MDIs based on conventional implant treatment is debatable. The lack of long-term studies and the differences in biological and mechanical properties of both implants make them not equal [23–25].

The number of MDIs was investigated in the literature concerning their biological complications and survival [18, 19, 26, 27]. On the other hand, regarding mechanical complications, fewer studies existed [28]. Therefore, there is little evidence of a consensus to guide the prosthetic treatment with MDIs regarding their number and influence on their mechanical performance [16]. Still, some companies suggest a number of MDIs for some cases depending on loading timing or patient age, but there is insufficient clinical evidence for those recommendations. As a result, it is common for some clinicians to follow conventional implant protocols while others modify the treatment according to their clinical experience.

Evaluating the *in vitro* retention of overdenture retained by different MDI numbers will help guide dentists regarding MDI treatment planning. This study includes an assessment of the effect of two, three, and four MDI attachments on overdenture retention loss and fatigue resistance following one year of simulated placement/removal. It also provides more information regarding the wear of MDI attachment inserts with respect to the MDI number. The first null hypothesis was that MDI number does not affect the *in vitro* retention of MDI retained overdenture placed in the atrophic mandible. The second null hypothesis was that the MDI number did not affect attachment insert wear following one year of simulated placement/removal.

## 2. Material and Methods

**Figure 1:**
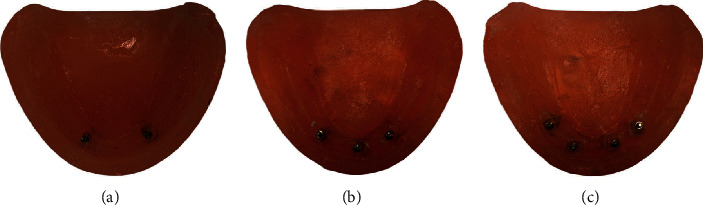
The distribution of the MDIs within the tested casts: (a) two MDIs at the canine area, (b) three MDIs (one at midline and two at canine area), and (c) 2 bilateral lateral incisors and two bilateral at 1st premolar area.

Nine testing models simulating atrophic mandible with no undercuts made from Orthoresin (Dentsply Sirona) were used to hold 27 ILZ MDIs from Southern Implants (13 mm (length) × 2.4 mm (width)). The MDIs were placed into each cast using a Ney surveyor to ensure parallel placement. Group A consists of two MDIs distributed at an interimplant distance of 23 mm, mimicking the canine area [29]. Group B consists of 3 MDIs, one at midline and two bilateral canine areas. For group C, the 4 MDIs were placed by subtracting 2 mm for each location, the interimplant distance was estimated at 19 mm representing the lateral incisors, and by adding 2 mm, the distance between the 1st premolar was calculated at 27 mm (2 bilateral lateral incisors and two bilateral at 1st premolar region), as shown in Figure 1.

**Figure 2:**
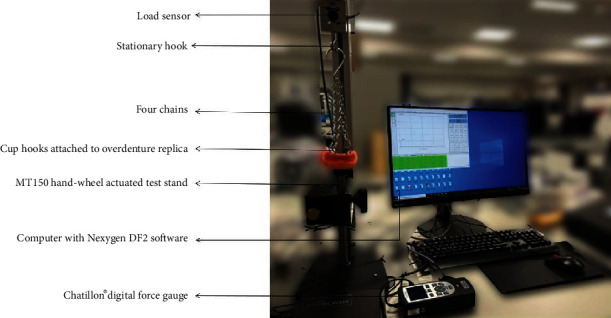
The testing apparatus, which includes the testing stand with sample holder with the sample, load sensor, force gauge, and monitor.

Acrylic overdenture replica containing MDI attachments (Rhein83 microstandard) was made for each cast. Four cup hooks were placed at the canine, and the first molar regions were incorporated into the acrylic, connected with four chains joined by pivoting joint for even pulling [30]. The joint was attached to a loading sensor at the top of a testing stand. The sensor is connected to a Chatillon digital force series II remote nondedicated gauge (DFS II-R-ND) to measure the peak load required for attachment dislodgment (Figure 2).

Fatigue test of overdentures was carried out by placement, and removal cycles were carried out along the MDI's long axis. The retention force (N) measured as the peak load during attachment break was graphically recorded and analyzed using the Nexygen DF V2 software.

The value was measured at the first cycle and then following completion of each 90-cycle simulating one month until completing 1080 placement/removal cycles simulating one year of use, three cycles per day assuming that an overdenture is expected to be removed three times a day for cleaning. The dislodgment rate was kept at one cycle per 3 seconds to allow nylon attachment elastic recovery [31].

Each group's mean of retentive force was calculated and analyzed using SPSS with a 95% confidence level using one-way ANOVA (*p* < 0.05). The significant differences among the groups were determined using Tukey's post hoc test. The percentage of retention force loss for each interimplant sample was also calculated using the following equation:

(1) $$\begin{array}{c} \text{Retention force loss }\left(\%\right)=\frac{\text{Initial Retention Force}-\text{Final Retention Force}}{\text{Initial Retentive Force}}\times 100. \end{array}$$

A paired *t*-test was used to compare the mean of the initial retentive force and the retentive force measured following one year of simulated placement/removal for each group (*p* < 0.05).

### 2.1. Examination of Attachment Wear

The inserts were examined using a light microscope before incorporating them within the overdenture to assess the inner diameter of the ring. Following completion of placement/removal testing, the inserts were removed from each overdenture and reexamined under a light microscope ECHO (Revolve, Bico Company, San Diego, USA). The images were interpreted using the built-in scale annotation of the microscope. The diameter measurements were recorded two times, two weeks apart, to evaluate intraexaminer reliability. The first measurement was analyzed when the intraexaminer reliability was confirmed using Cohen's kappa coefficient. The mean diameter of the used retentive insert in each group was compared to the mean diameter before testing using paired *t*-test (*p* < 0.05). On the other hand, the diameter differences between groups were identified using one-way ANOVA tests (*p* < 0.05). In the event of significant differences between the diameter means, Tukey's post hoc multiple comparison tests were used (*p* < 0.05).

## 3. Results

### 3.1. Comparison of Retention Forces between Groups

**Figure 3:**
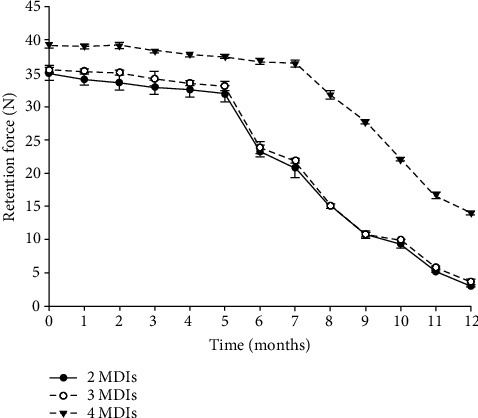
Mean retention force values for samples measured at each month for one year and the mean value for each group.

During testing, the measurements were recorded for each tested sample presented in Figure 3.

At first, there was a significant difference between groups using ANOVA for the values measured at the first cycle. The Tukey post hoc test showed no significant difference between 2 and 3 (Table 1), while the retention force provided by 4 MDIs was significantly higher than 2 and 3 MDIs with *p* values of 0.001 and 0.002, respectively. Similar results were recorded during the following months (Table 2).

Following completion of placement and removal simulating one year of function (1080 cycles), similar results were also found. There was a significant difference between groups using a one-way analysis of variance with *p* ≤ 0.001. At the same time, Tukey's post hoc test showed no significant difference between 2 and 3 (*p* = 0.21), while the retention force provided by 4 MDIs was significantly higher than 2 and 3 MDIs with *p* values ≤ 0.001 and ≤ 0.001, respectively.

### 3.2. Comparison of Retention Forces Pre- and Postsimulated Placement/Removal

A dependent *t*-test of the pre- and postloading retention forces was used to assess the retention loss. The analysis showed a statistical significance in the measured retention pre- and postloading in all groups. The *p* values were ≤0.001 for the 2 MDI group, ≤0.001 for the 3 MDI group, and ≤0.001 for the 4 MDI group. Therefore, following one year of overdenture placement/removal, the retention of the attachment significantly reduces regardless of MDI number.

### 3.3. Percentage of Retention Loss

**Figure 4:**
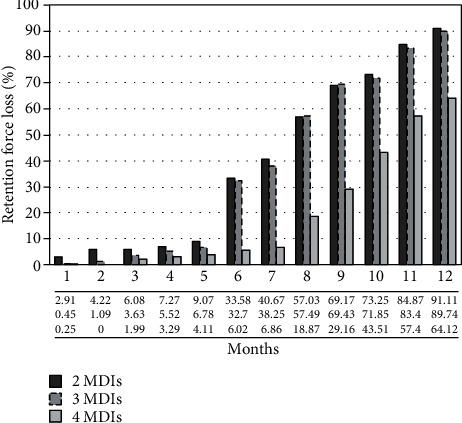
The percentage of retention loss among the groups following one year of testing.

Measurements of the retention loss pre and post one year of simulated placement/removal are shown in Figure 4.

### 3.4. Comparison of Insert Wear

Before placing the attachments within the overdenture replica, the inner ring diameter of the inserts was measured using a light microscope. Similarly, the retentive insert diameter measurement was carried out for each retrieved attachment following the testing period completion. Two diameter measurements were taken each time the inserts were examined by one examiner two weeks apart (Table 3).

In order to assess the reliability of the examiner, the diameter of the inner insert was measured twice pre- and posttest. Cohen's kappa index was calculated for both pre- and posttest measurements. The mean diameter of the new retentive insert pretest was 1.79 mm ± 0.01, very close to 1.8 mm diameter per manufacturer description. The kappa value of the diameter of the new insert in each group was about 0.73 for group A, 0.82 for group B, and 0.85 for group C which shows high agreement and hence reliable measurements.

For the used attachment inserts (posttesting), the intraexaminer reliability was also calculated at 0.79 for group A, 0.87 for group B, and 0.80 for group C, which also shows high agreement between the readings.

A dependent *t*-test was carried out to compare the means measured pre- and posttesting for each group. There was a significant difference between the diameters of the new retentive inserts and all tested groups. For the 2 MDIs, *p* value was ≤0.001, ≤0.001 for 3 MDIs, and ≤0.001 for 4 MDIs. Such results show that abrasion and compression wear resulted in the diameter increase within the insert surface following simulated one year of placement/removal.

A one-way analysis of variance was used to compare the postloading diameter to compare the diameter changes between all groups. A statistical difference in diameter was measured between the three groups (*p* ≤ 0.001). Using the Tukey post hoc test, no significant difference was found between the mean diameters measured for 2 and 3 MDI models (*p* = 0.61). In contrast, the 4 MDI model showed a significant difference in mean diameter compared to both the 2 and 3 MDI models with *p* ≤ 0.001 and *p* ≤ 0.001, respectively.

**Figure 5:**
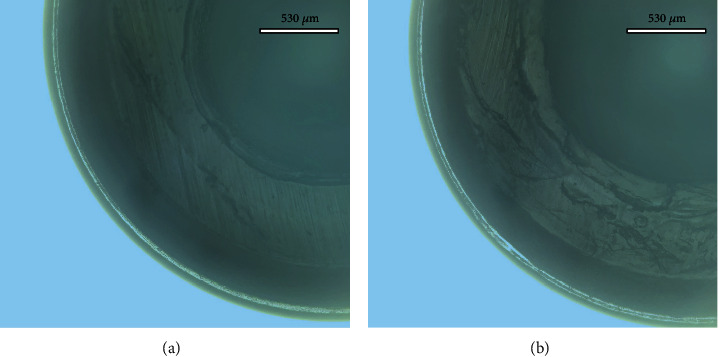
Images of pretest (a) and posttest (b) of the same insert examine the same area to show the abrasion wear changes in the surface texture of the inner ring marked in solid in pretest and dashed in posttest, compression lines showing on the peripheral nylon rim.

The microscopic examination of the new inserts showed a uniformly round inner ring with no signs of tears at the periphery. All retrieved inserts following testing showed signs of deformation within the inner ring in the form of compression and abrasion wear (Figure 5). The nylon inserts were kept in the metal housing to avoid further damage when attempts were made to remove them. However, it was noticeable that the diameter of the inserts used for the 4 MDI models had less loss of surface, i.e., a smaller diameter than the 2 and 3 MDI inserts.

## 4. Discussion

Overdenture retention is necessary for patients' satisfaction with their prostheses [32]. However, adequate overdenture retention is debatable, and many studies reported different values of adequate retention [33–35]. The retentive force should be high enough to prevent displacement during the function to provide comfort; however, it should not interfere with the patient removing the prosthesis [36]. Therefore, considering the patient's manual dexterity should be a factor in deciding the number of implants regarding retention. This is particularly essential when treating elderly patients targeted for the MDI treatment approach [37].

For overdenture, the placement of two implants is a standard of care [1, 2]. Nevertheless, the placement of more implants was associated with increased overdenture retention and reduced rotation [38–40]. Similarly, the results of this study showed that 4 MDIs provided more retention and less retention loss than 3 and 2 MDIs; therefore, the first null hypothesis was rejected. This agrees with clinical results on patients' satisfaction with overdenture regarding increasing the MDIs [41]. However, increasing MDI number was not always associated with increased resistance to retention loss as there was no difference in the results between 2 and 3 MDIs. This can be related to the axial direction of the dislodgment, the small size of the MDI attachment, or the proximity of the midline MDI to the canine area where the second and third MDIs were placed. In a study that utilized the same sample design and tested the same attachments, 4 MDIs showed higher resistance to para-axial dislodgement. Moreover, the wide distribution of 3 and 2 MDIs showed an improvement in resisting such forces [20]. Therefore, more studies should assess the effect of MDI distribution on overdenture retention.

The values measured following one year of testing showed that 4 MDI models lost about 1/3 of their initial retention following nine months. At the same time, the 2 and 3 MDI models lost about 1/3 of their retention following only six months of testing. Furthermore, at the end of testing cycles, both 2 and 3 MDI models retained about 10% of their initial retention force, while the 4 MDI models retained about 36% of their retentiveness. Higher initial retentive forces were also recorded for the 4 MDI group with a mean of 39.16 N, while the mean retentive force measured following six months of testing was about 36.80 N. In comparison to O-ring, the retentive force of 4 MDI with O-rings at vertical dislodgment was about 27.34 N before loading and 14.55 N after simulating six months of placement and removal [42]. However, the O-rings last about 6 to 9 months until they are replaced [43] compared to 12 months of nylon inserts tested [44].

Due to the little evidence on MDI overdenture, several researchers have attempted to compare them to conventional implants. All three groups showed adequate overdenture retention within the testing period based on values obtained from conventional attachments from the literature regarding satisfactory retention [33, 45].

Comparable results were found between four MDIs and two conventional implants regarding overdenture success and patient satisfaction [5, 14]. Therefore, the overdenture retained by two or four MDIs was considered an alternative treatment option for two conventional implants [41]. This treatment was advantageous as patients with two MDIs have less postoperative pain and fewer complications than those with two conventional implants [22]. However, the outcome of both treatment approaches mentioned for MDI numbers is not extensively studied, *in vitro* or *in vivo*, as with conventional implants [33, 46]. Indeed, a treatment option that includes two MDIs will cost less than two conventional implants due to the price difference [22]. Subsequently, increasing the MDI number will increase the price of the treatment and maintenance. Based on this study, due to the lack of significant benefit in the overdenture retention from adding a third MDI, less cost and fewer risks of surgical complications are achievable with 2 rather than 3 MDIs.

Utilizing two implants rather than 4 to retain and stabilize an overdenture was suggested as a treatment option for atrophic mandibles [11]. However, that cannot always be the case, and the need for MDIs to retain an overdenture on the atrophic mandible where the residual ridge is compromised is an option [8]. In residual atrophic ridges, bone loss and ridge configuration limit the implant positioning [12]. A lingual undercut at the anterior mandible can increase the risk of perforation to the lingual bone during placement [47]. As a result, injury to the incisive blood vessels can lead to life-threatening complications [48–51]. The mental foramen and inferior alveolar nerve, or its branches, also pose a risk of injury at the interforaminal area. Therefore, placement of more implants may require positioning them around those areas increasing the risk of surgical complications.

The manufacturer of the nylon insert used in this study recommended changing them annually [44]. That was apparent as the retentive force of 4 MDIs measured following six months of loading [42] was similar to the mean retentive force of 14.05 N recorded in this study by 4 MDIs following one year. The loss of retention following function is expected; hence, many studies reported a reduction of retention force with increasing the number of testing cycles [33, 52–54]. Thus, all MDI attachments exhibited retention loss following testing despite the MDI number; therefore, the second null hypothesis was rejected. The 2 MDI models exhibited a loss of 91% of their initial measured retention, while the three and four MDI models lost about 89.74% and 64.12%, respectively. This reduction in retention force under function is due to attachment wear during placement/removal [55, 56].

Attachment wear was investigated in several observational studies using microscopic examination of insert diameter following placement/removal [52, 57, 58]. Following one year of testing, all retrieved inserts showed an increased diameter of their original measured inner ring. Nevertheless, the diameter of the 4 MDIs was the least affected by this increase as the difference between the pre- and postloading diameters was about 0.24 mm compared to 0.35 mm and 0.37 mm in the 2 and 3 MDI models, respectively. One study investigated the retention of overdenture retained by four MDIs placed at the interforaminal area. They found that changes in retentive forces of O-rings were more evident in posterior implants rather than anterior MDIs [28]. In this study, the diameter changes were similar within each model as the wear was not affected by the attachment location. However, based on the results, the placement of 4 MDIs might reduce the need for annual insert replacement.

## 5. Conclusion

Within the limitation of this study, the retention of the attachment reduces following one year of function regardless of MDI number. However, the retention force and attachment wear of MDI retained overdenture placed in atrophic mandible could improve with increasing the MDI number to 4. However, there was no difference in retention force or attachment wear when 2 or 3 MDI overdentures were compared. Therefore, 2 MDI overdenture can present comparable retention for mandibular overdenture while offering less risk of surgical complications and a more economical solution for patients instead of 3 MDI overdenture.

## Figures and Tables

**Table 1:** Tukey's post hoc test showed no significant difference between 2 and 3.

Mean of retention force for 2 and 3 MDI models	ANOVA test sig.
At one month	0.14
At 2 months	0.08
At 3 months	0.27
At 4 months	0.27
At 5 months	0.27
At 6 months	0.63
At 7 months	0.35
At 8 months	0.99
At 9 months	0.997
At 10 months	0.24
At 11 months	0.07

**Table 2:** The retention force provided by 4 MDIs compared to 2 and 3 MDIs using ANOVA and post hoc tests where the mean difference is significant at the 0.05 level^∗^.

ANOVA test sig.	Mean of retention force measured at	4 MDI model in comparison to	Tukey post hoc test sig.
≤0.001^∗^	1 month	2 MDI model	≤0.001^∗^
		3 MDI model	≤0.001^∗^
≤0.001^∗^	2 months	2 MDI model	≤0.001^∗^
		3 MDI model	≤0.001^∗^
≤0.001^∗^	3 months	2 MDI model	≤0.001^∗^
		3 MDI model	≤0.001^∗^
≤0.001^∗^	4 months	2 MDI model	≤0.001^∗^
		3 MDI model	≤0.001^∗^
≤0.001^∗^	5 months	2 MDI model	≤0.001^∗^
		3 MDI model	≤0.001^∗^
≤0.001^∗^	6 months	2 MDI model	≤0.001^∗^
		3 MDI model	≤0.001^∗^
≤0.001^∗^	7 months	2 MDI model	≤0.001^∗^
		3 MDI model	≤0.001^∗^
≤0.001^∗^	8 months	2 MDI model	≤0.001^∗^
		3 MDI model	≤0.001^∗^
≤0.001^∗^	9 months	2 MDI model	≤0.001^∗^
		3 MDI model	≤0.001^∗^
≤0.001^∗^	10 months	2 MDI model	≤0.001^∗^
		3 MDI model	≤0.001^∗^
≤0.001^∗^	11 months	2 MDI model	≤0.001^∗^
		3 MDI model	≤0.001^∗^

**Table 3:** The mean of the inner ring diameter of all groups pre- and posttesting, read 2, measured two weeks after the first reading.

Groups	Pretest inner ring diameter (mm)		Posttest inner ring diameter (mm)	
	Read 1	Read 2	Read 1	Read 2
2 MDIs	1.79 mm ± 0.000	1.79 mm ± 0.01	2.15 ± 0.43	2.15 ± 0.42
3 MDIs	1.79 mm ± 0.000	1.80 mm ± 0.000	2.17 ± 0.025	2.16 ± 0.027
4 MDIs	1.79 mm ± 0.01	1.79 mm ± 0.02	2.04 ± 0.33	2.04 ± 0.33

## Data Availability

All data are presented within the article.
